# Effect of actin C-terminal modification on tropomyosin isoforms binding and thin filament regulation

**DOI:** 10.1016/j.bbapap.2008.10.014

**Published:** 2009-02

**Authors:** Radosław Skórzewski, Małgorzata Śliwińska, Danuta Borys, Apolinary Sobieszek, Joanna Moraczewska

**Affiliations:** a Kazimierz Wielki University in Bydgoszcz, Department of Experimental Biology, Chodkiewicza 30, 85-064 Bydgoszcz, Poland; b Institute for Biomedical Aging Research, Austrian Academy of Science, Life Science Center, Mitterweg 24, A-6020 Innsbruck, Austria

**Keywords:** Actin, Truncated actin, Tropomyosin, Smooth muscle, Non-muscle, Regulation

## Abstract

Tropomyosins, a family of actin-binding regulatory proteins, are present in muscle and non-muscle cells. Multiple tropomyosin (TM) isoforms differ in actin affinity and regulatory properties, but little is known about the molecular bases of these differences. The C-terminus of actin stabilizes contacts between actin subunits in the filament and interacts with myosin and regulatory proteins. The goal of this work was to reveal how structural changes in actin and differences between TM isoforms affect binding between these proteins and affect thin filament regulation. Actin proteolytically truncated by three C-terminal amino acids exhibited 1.2–1.5 fold reduced affinity for non-muscle and smooth muscle tropomyosin isoforms. The truncation increased the cooperativity of myosin S1-induced tropomyosin binding for short tropomyosins (TM5a and TM1b9a), but it was neutral for long isoforms (smTM and TM2). Actin modification affected regulation of actomyosin ATPase activity in the presence of all tropomyosins by shifting the filament into a more active state. We conclude that the integrity of the actin C-terminus is important for actin–tropomyosin interactions, however the increased affinity of tropomyosin binding in the S1-induced state of the filament appears not to be involved in the tropomyosin isoform-dependent mechanism of the actomyosin ATPase activation.

## 1 Introduction

Tropomyosins (TMs) are a family of regulatory proteins closely associated with the actin filament. All TMs are two-chained α-helical coiled-coil proteins that bind along both sides of the actin filament. While bound to F-actin TM molecules form long, continuous strands, due to strong end-to-end interactions. Tropomyosins control muscle contraction and motility of non-muscle cells through regulation of the thin filament interactions with myosin motors and actin-binding proteins. The isoforms bind to actin with a wide range of affinities and exhibit different regulatory mechanisms for actin activated myosin ATPase [1–5].

During development TM isoforms are differentially expressed and sorted to specific cellular compartments. The mechanism of isoform sorting is complicated and not well understood. Gunning and colleagues [4,5] suggested that the isoforms are driven to their final cellular destinations by affinity of TMs for specific actin structures. Studies on actin modified by site-directed mutagenesis [6–9] or limited proteolysis [10] revealed that the interactions between TM and actin involve direct contacts with charged surface residues as well as allosteric structural transitions within actin itself. It is well established that the affinity of TMs for actin is defined by isoform-specific sequences of TM encoded by alternative exons (reviewed in [1,3]). Particularly important are the ends, as the end-to-end overlap greatly increases affinity of TMs for actin [11–13]. The process of thin filament assembly is complex and cooperative. Initial binding of TM molecules to an isolated site on the actin filament, where no TM–TM contacts take place, is followed by binding to singly and doubly contiguous binding sites, with both TM termini forming end-to-end contacts except at the filament ends [11]. An increase in the affinity occurs when TM binds to an adjacent site and it is thought to be caused by direct contacts between adjacent TM molecules and by conformational changes within actin itself [14]. However, the mechanism of TM–actin interaction is not fully understood.

Using actin proteolytically modified by selective removal of three C-terminal amino acids (actin_−3_), we have recently shown that distortion of the actin structure within the C-terminal segment affects actin interactions with skeletal α-TM, but the effect differs depending on the state of the thin filament activation [15]. The analysis was done in three thin filament activation states — blocked, closed and open, which were proposed by McKillop and Geeves [16] on the basis of the effect that TM and troponin (± Ca^2+^) exert on myosin binding to the regulated thin filament. In striated muscle the catalytic states correlate well with structural states visualized by three-dimensional reconstructions of electron microscopic images of the thin filaments, in agreement with the steric blocking mechanism of actin–myosin regulation [17,18]. However, TM isoforms specific for smooth muscle and non-muscle cells do not always follow the same pattern in terms of the function–structure relationships. For example, smooth muscle TM was a better activator of actin-activated myosin S1 ATPase than skeletal muscle TM [19–21], but its location on actin was closer to a position which for skeletal TM was defined as the blocked state [22]. This shows that in case of smooth muscle TM steric blocking cannot explain the mechanism of actin filament regulation [21].

The actin C-terminus is an important region of the molecule involved in forming an interface between actin monomers within the filament and between actin and several actin-binding proteins (reviewed in [23]). Conformational changes in this region can be transmitted to distant regions of the molecule and affect interactions with TM. Local and distant effects of the C-terminal truncation on actin filament structure and dynamics are well characterized [24–26]. Therefore we used truncated actin as a model of conformationally altered actin to study the binding modes of TM isoforms in thin filament activation states. To examine the effects of actin conformational changes from the TM point of view, we selected TMs with isoform-dependent sequence combinations. We observed that modification of actin slightly affected parameters of TMs binding to actin alone, but only short TM isoforms binding was affected by actin truncation when the filament was activated by rigor-bound myosin heads. This indicates that structural changes associated with actin modification can modulate interactions with TM in isoform-dependent manner. This might underly the mechanism of TM isoforms sorting to different cellular compartments. However, regulation of actomyosin ATPase by TM isoforms does not seem to depend on the affinity of TMs to actin in the activated state. We conclude that in the presence of TM the actin C-terminus is involved in maintaining the filament in an inhibited state. Once the C-terminus is removed, the filament is shifted into a more active state. Preliminary results of this work were reported earlier [27].

## 2 Materials and methods

### 2.1 Protein preparation

Actin was isolated from chicken pectoral muscle acetone powder according to the method of Spudich and Watt [28]. Actin_−3_, devoid of the three C-terminal amino acids, was prepared by trypsinolysis of G-actin in which tightly bound Ca^2+^had been exchanged for Mg^2+^[25]. This method allows for specific removal of three residues (Lys^373^, Cys^374^ and Phe^375^) from the actin C-terminus.

**Fig. 1:**
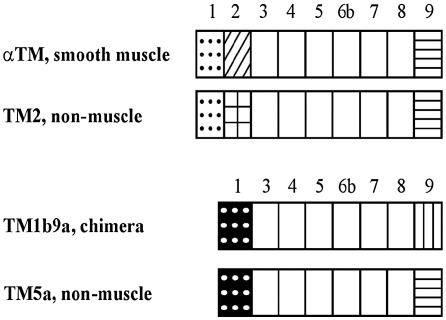
Schematic illustration of the α-TM variants used in this work. All isoforms are expressed in smooth muscle and non-muscle cells except for the chimeric TM (TM1b9a) for which no natural counterpart has been found. The TM1b9a differs from TM5a only in the sequence of C-terminus that is encoded by striated muscle-specific exon 9a. Boxes represent TM regions encoded by exons labeled respectively on top of the schemes. The proteins with N-termini encoded by exons 1a and 1b belong respectively to long and short classes of TM. The C-terminal sequences are encoded either by striated muscle specific exon 9a or constitutive exon 9d. Third region of difference is encoded either by constitutive exon 2b or smooth muscle-specific 2a. The alternative exons expressed in TM isoforms are represented by filled boxes (exon 1a-white dotted, 1b-black dotted, 2a-hatched, 2b-checked, 9a-vertical bars, 9d-horizontal bars).

The four tropomyosin isoforms selected for this study were products of the rat α-TM gene (*TPM1*). Regions of sequence differences are described in the Fig. 1 legend. The isoforms were expressed in *E. coli* strain BL21(DE3) transformed with rat α-TM cDNA cloned into pET11d (Novagene, Houston, TX), as described in [29]. The cDNA clones were the gift of Dr. S. Hitchcock-DeGregori. The proteins were purified from bacteria according to the protocol described earlier [29]. All isoforms were unacetylated at the N terminus.

Myosin S1 was prepared by papain digestion of chicken pectoral muscle myosin according to Margossian and Lowey [30].

The concentration of actin was determined spectrophotometrically at 290 nm using an extinction coefficient of 0.63 for 0.1% actin, MW 42,000. The concentration of C-terminally truncated actin was determined densitometrically as described in [15]. Myosin S1 concentration was calculated from an extinction coefficient of 0.83 at 280 nm (0.1%) and protein MW 130,000. Concentrations of recombinant TMs were determined by the differential absorption spectra of tyrosine (refs. in [29]).

### 2.2 Actin binding assay

Tropomyosin binding to actin was studied by a cosedimentation assay as described earlier [29] with modifications as in [15]. The assay samples were composed of 5 μM filamentous F-Mg-actin and TM at concentrations varying from 0.2 to 10 μM. For smTM, TM2 and TM1b9a actin binding buffer contained 5 mM imidazole, pH 7.0, 1 mM DTT, 150 mM NaCl and 2 mM MgCl_2_. Because TM5a binds very strongly to actin [29], actin binding of this isoform was difficult to measure in the above conditions. To make the analysis possible, the concentrations of NaCl and MgCl_2_ were reduced to 30 and 0.5 mM, respectively. Protein mixtures were centrifuged for 1 h at 40,000 rpm in Beckman rotor 42.2. The composition of the proteins in pellets and the amount of free TM left in supernatant were examined on 10% SDS-PAGE. The gels stained with Coomassie Blue were scanned with Plustek OpticPro ST12 and quantitated using EasyDens (Cortex Nova, Bydgoszcz, Poland) software. The TM bound to actin was calculated as the TM/actin band intensity ratio normalized to the maximum ratio reached at saturation. The concentration of unbound TM was calculated from intensities of TM in supernatant using a standard curve constructed from the same TM isoform. The apparent association constant (*K*_app_) of TM to actin was calculated by fitting the experimental data to Hill equation (Eq. (1)) in SigmaPlot (Systat Software Inc. San Jose, CA).

(1) $$v=n{\left[TM\right]}^{\alpha^{H}}K_{\text{app}}/1+{\left[TM\right]}^{\alpha^{H}}K_{\text{app}}^{\alpha^{H}}$$

where *v* = fraction maximal TM binding to actin, *n* = maximal TM bound, [TM] = free [TM], and *α*^*H*^ = cooperativity coefficient.

**Fig. 2:**
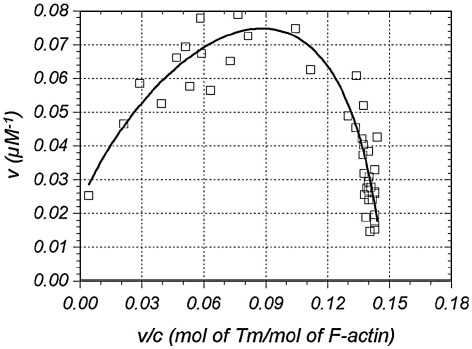
Binding of smTM to native F-actin and evaluation of the association constant *K*_o_, cooperativity *ω*, and binding stoichiometry n, using McGhee and von Hippel approach. Solid line represents the binding function (2) computed with the optimal parameters obtained from non-linear regression analysis. *v* is the number of mol of tropomyosin bound per mol of actin, *c* is the concentration of free tropomyosin. For details see Materials and methods.

The McGhee and von Hippel equation (Eq. (2)) was also used to calculate the equilibrium constant for the association of TM with an isolated binding site (*K*_0_) and equilibrium constant for moving TM from an isolated binding site to a singly contiguous binding site (*ω*), as described by the authors [31]^1^.

(2) $$v/c=K_{0}\left(1-nv\right){\left(\frac{(2\omega -1)(1-nv)+v-R}{2(\omega -1)(1-nv)}\right)}^{n-1}{\left(\frac{1-(n+1)v+R}{2(1-nv)}\right)}^{2}$$

where,

*R* = {[1 − (*n* + 1) *v*]^2^ + 4 *ωv* (1 − *nv*)}^1/2^; *v* = the number of mol of TM bound per mol of actin, *n* = stoichiometry of TM binding to actin, and *c* = the concentration of free TM. The binding data were analyzed by fitting the experimental points to the Eq. (2) using non-linear regression software Scientists from MicroMath Scientific Software Inc., (Saint Louis, Mo) in which χ^2^-function between experimental and theoretical values of a mathematical model is minimized. The character of the curve generated by the Eq. (2) is shown in Fig. 2.

### 2.3 S1-induced binding of TM to actin

Tropomyosin binding to actin induced by myosin S1 was analyzed by cosedimentation as described in [15]. Samples of 5 μM F-Mg-actin and 1 μM TM were titrated with S1 added to final concentrations between 0 and 6 μM. TMs were assayed under conditions unfavourable for binding of TM to actin alone: 5 mM imidazole pH 7.0, 30 mM NaCl, 0.5 mM MgCl_2_ and 1 mM DTT. In the presence of TM5a, the NaCl concentration was lowered to 12 mM. After centrifugation (1 h at 40,000 rpm, 20 °C in Beckman rotor 42.2), the proteins cosedimented with F-actin were separated by electrophoresis on 12% SDS gels and analyzed densitometrically. Saturation curves were plotted as TM/actin and S1/actin bands intensity ratios, normalized to the values reached at maximal binding, versus the S1/actin molar ratio. The number of S1 bound per actin that was required for half maximal saturation of actin filament with TM was calculated by fitting in SigmaPlot modified Hill equation to the experimental data:

(3) $$v=\left(n{\left[X\right]}^{\alpha^{H}}K_{app}^{\alpha^{H}}/1+{\left[X\right]}^{\alpha^{H}}K_{app}^{\alpha^{H}}\right)+C$$

where *v* = TM/actin density ratio, [X] = S1/actin molar ratio, *C* = TM/actin density ratio at 0 S1 concentration. The S1/actin molar ratio required for half maximal saturation of actin with TM was calculated as 1 / *K*. The maximal TM/actin ratio was the same for both F-actin_−3_ and native actin, indicative of complete saturation.

### 2.4 Actomyosin MgATPase

Myosin S1 ATPase activity was measured in 30 mM NaCl, 5 mM imidazole, pH 7.0, 2 mM MgCl_2_, 1 mM DTT at 0.38 μM S1with actin concentrations varying between 5 and 20 μM. TM, when present, was added to actin stock solution at equal molar concentrations. The reaction was initiated by adding MgATP to 5 mM and stopped after 20 min. with 3.3% SDS and 30 mM EDTA. The amount of liberated phosphate was determined colorimetrically [32].

## 3 Results

### 3.1 Binding of TM isoforms to actin

To examine whether conformational changes in the C-terminal region of actin influence binding of TM isoforms, we compared affinities of α-TM isoforms to native and C-terminal truncated actin in direct cosedimentation assay. Since differences in sequence among the isoforms are limited to well defined regions (Fig. 1), we assumed that any significant differences between native actin and actin_−3_ binding would reveal recognition of the specific TM sequence by actin.

**Fig. 3:**
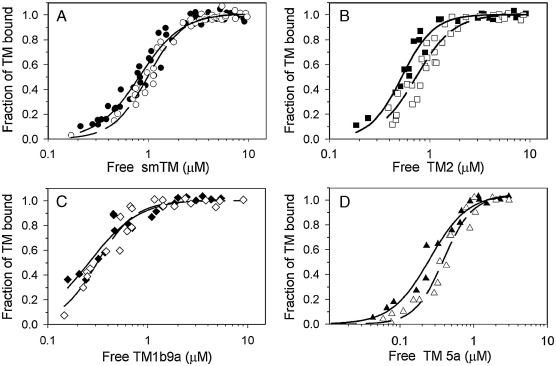
Effect of actin truncation on its affinity for tropomyosin isoforms. Binding of smTM (A), TM2 (B), TM1b9a (C), and TM5a (D) to native F-actin (closed symbols) or F-actin_−3_ (open symbols) was assayed as described in Materials and methods. Conditions: 5 mM imidazole, 1 mM DTT, 150 mM NaCl, 2 mM MgCl_2_, with pH adjusted to 7.0, in the case of D) concentrations of NaCl and MgCl_2_ were respectively 30 and 0.5 mM. Data are from 3 to 5 independent experiments. Binding curves were drawn by fitting the experimental points to Hill equation (Eq. 1); (solid line) native F-actin; (dashed line) F-actin_−3_.

Removal of three residues from actin C-teminus had a small effect on the binding of each of the TM isoforms used (Fig. 3). The equilibrium binding constants (*K*_app_) obtained by fitting the experimental points to the Hill equation (Eq. 1) are summarized in Table 1 and show that actin truncation reduced the isoforms' affinity by a factor ranging between 1.2 and 1.5. The cooperativity coefficient (*α*^*H*^) of TMs binding to F-actin_−3_ was slightly greater than that for the binding to F-actin. The only TM isoform which bound to F-actin_−3_ with lower cooperativity was TM2 (Table 1). This might suggest that actin modification affects the affinity of TM either through changing direct TM–actin contacts or through distortion of binding cooperativity.

Adapting the McGhee and von Hippel model for ligand binding to a linear lattice [33], we analyzed the effect of actin truncation on TM affinity to an isolated binding site and an equilibrium constant for moving TM from an isolated binding site to a singly contiguous binding site, which is a measure of binding cooperativity [11,31,34]. The analysis revealed that in the case of three isoforms (smTM, TM1b9a and TM5a) the affinity to an isolated binding site (*K*_o_) was reduced and binding to a contiguous site (*ω*) was increased by modification of the actin C-terminus. The TM2 bound to truncated actin with a binding coefficient *K*_o_ similar to that of native actin and with the lower cooperativity (Table 1). Assuming that binding to an isolated site reflects direct TM–actin contacts and cooperativity coefficient reflects long-range interactions induced by the TM that is already bound, our results suggest the C-terminal region can affect the binding of tropomyosins through both direct contacts and/or long-range interactions. We then asked whether the removal of C-terminal residues affects also TM binding in the fully active myosin-induced state and, if it does, what is the response of TM isoforms to the actin modification.

### 3.2 S1-induced binding of TM isoforms

The strong, rigor binding of myosin heads to actin is required for full activation of the thin filament [16]. In the activated state the affinity of TM for actin strongly increases [35,36]. The cooperativity of the myosin S1-induced shift of the filament into the active state can be assessed in an assay developed by Eaton [35], in which TM binding to actin is analyzed under conditions in which binding of TM alone is poor, but it increases upon the binding of myosin heads to actin. It is thought that S1 induces conformational change in actin, which facilitates binding of TM with high affinity. The lower the number of rigor-bound S1 that are required for actin saturation with TM, the greater the cooperativity [29].

**Fig. 4:**
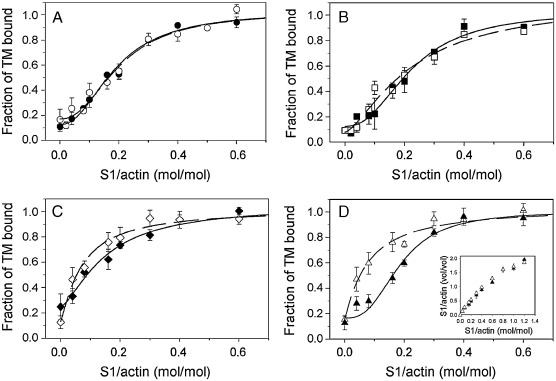
Myosin S1-induced binding of TM to native and truncated actin. Binding of smTM (A), TM2 (B), TM1b9a (C), and TM5a (D) to native F-actin (closed symbols) and F-actin_−3_ (open symbols) was assayed under the following conditions: 5 mM imidazole, pH 7.0, 30 mM (or 12 mM for TM5a) NaCl, 0.5 mM MgCl_2_, 1 mM DTT. S1/actin is a volumetric myosin S1 and actin density ratio obtained from the pellets separated on SDS gels. Vertical bars represent standard errors. Inset represents binding of the myosin S1 to native F-actin (closed triangles) and F-actin_−3_ (open triangles).

Actin C-terminal modification had different effects on S1-induced binding of the long and short TM isoforms. While long isoforms (smTM and TM2) were not sensitive to actin truncation and bound to both types of actin with the same cooperativity, the short TMs, TM1b9a and TM5a, bound more easily to modified than to native actin (Fig. 4). The numbers given in Table 2 illustrate the cooperativity of myosin S1-induced TM binding. The cooperativity was obtained from non-linear regression fitting that averaged experimental points to Eq. 3 (see Materials and methods).

The S1 concentration for half-maximal saturation of F-actin_−3_ with TM5a was half that of native actin (*p* = 0.002). The binding of approximately one myosin head per segment of six actins (which interacts with one TM molecule) was necessary for full saturation of native actin with TM5a, whereas saturation of truncated actin was completed when only 0.5 myosin heads per six actins (or per one TM) were bound. The differences in S1-induced TM binding were not caused by altered S1 binding, because actin C-terminal modification did not affect S1 binding to actin (Fig. 4D, inset). The parameters of S1-induced binding of TM1b9a were similar to the parameters obtained for TM5a (Table 2). However, the statistical significance of the difference between native and truncated actin in the case of TM1b9a is ambiguous, as the *t*-test generated *p* = 0.07, which minimally exceeds the threshold of significance.

### 3.3 Regulation of actin-activated myosin S1 ATPase activity

Since the cooperativity with which myosin S1 induces TM binding to actin is a measure of the thin filament tendency to switch into the active state [29], one would expect that truncated actin complexed with short TMs would activate myosin S1 ATPase more readily than in complex with long TM isoforms. To verify this hypothesis we analyzed the effect of actin truncation on the regulation of actin-activated myosin S1 ATPase by tropomyosin isoforms.

**Fig. 5:**
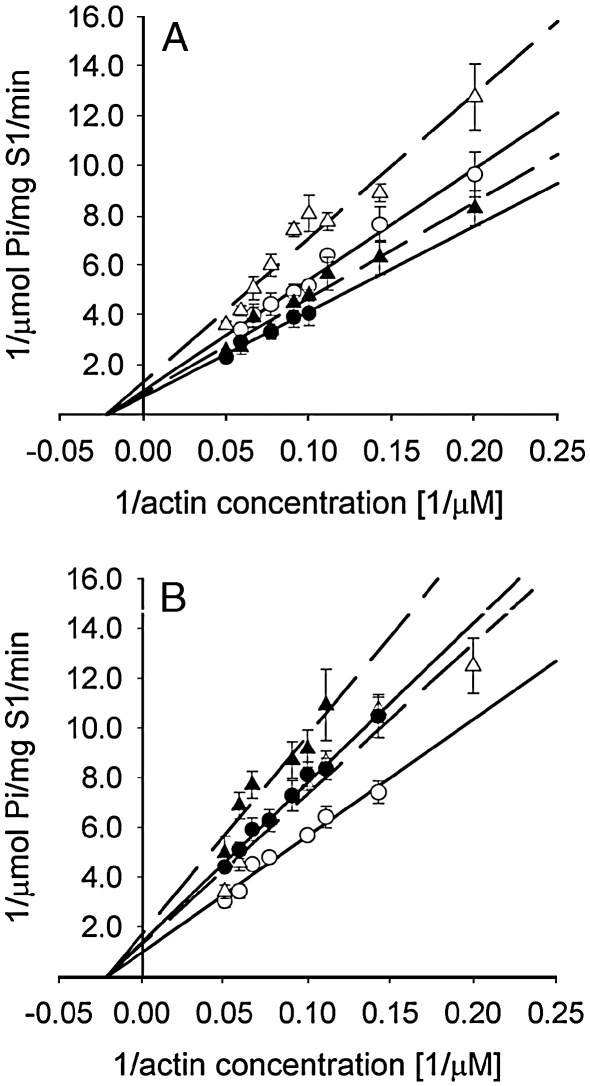
Double reciprocal plot of S1 MgATPase activity versus actin (open symbols) and actin–TM (closed symbols) concentration. The regulation by TM5a (A) and TM2 (B) in the presence of native F-actin (circles) and F-actin_−3_ (triangles) was measured in 5 mM imidazole pH 7.0, 30 mM NaCl, 2 mM MgCl_2_, 1 mM DTT at 0.38 μM S1. The data were averaged from 4 experiments. The lines show linear regressions for each set of averaged points.

The ATPase activity was analyzed at constant S1 concentration by increasing actin or actin–TM concentrations. Fig. 5 illustrates double reciprocal plots of actin-activated S1 ATPase activity for representative tropomyosins (TM5a and TM2). Because in the conditions used in this assay the S1 enzymatic activity was far below saturation, we used double reciprocal plots rather than saturation curves. This generated an elevated standard error for the calculated ATPase parameters, *K*_M_ and *V*_max_, despite the small experimental error in each measured point. In agreement with earlier observations [15,24], the removal of C-terminal residues from actin reduced maximal rate of S1 ATPase (*V*_max_), whereas the Michaelis–Menten constant (*K*_M_) was not affected (Table 3). Saturating the filaments with the TM isoforms did not change the *K*_M_. In contrast, the effects on *V*_max_ depended on the TM isoforms (Table 3). The TM5a activated the ATPase, which agrees with a previous report showing that this isoform is very efficient in the activation of thin filament [37]. The other TM isoforms (TM2, smTM and TM1b9a) inhibited the ATPase activity by lowering *V*_max_.

**Fig. 6:**
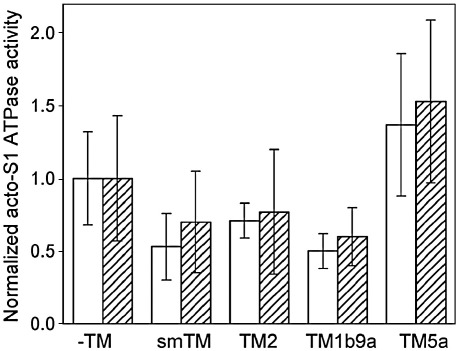
Effect of actin C-terminal truncation on the regulation of actin-activated myosin S1 MgATPase. The normalized acto-S1 ATPase activities are the maximal rates (*V*_max_) divided by the activity with unregulated native F-actin (open bars) or F-actin−3 (hatched bars). Error bars are SE.

In order to compare effects of the isoforms to regulate the ATPase in complexes with normal and modified actin, we normalized the data for each type of actin by dividing *V*_max_ observed in the presence of TM by *V*_max_ obtained for actin alone. The normalized activities, shown in Fig. 6, indicate that when complexed with F-actin_−3_, smTM, TM2 and TM1b9a inhibited the ATPase by a smaller degree than with native actin. In turn, the degree of activation of the ATPase observed in the presence of TM5a was grater for F-actin_−3_ than for native actin. However, the differences between the short and long isoforms in the degree of inhibition (or activation in the presence of TM5a) were not significant. This indicates that actin affinity detected for short TM isoforms in the S1-induced TM binding assay is not the major determinant in the regulation of active cross-bridges.

## 4 Discussion

The purpose of this work was to elucidate the specific mechanism(s) of TM-dependent thin filament regulation. We investigated the role that the actin C-terminal region plays in interactions with TM isoforms and in the regulation of cross-bridge cycling. Because properties of actin devoid of three C-terminal residues are well characterized, we used this modification to study interactions between actin and TM, the two major components of the thin filament.

In actin the C-terminal segment is located in subdomain 1. In the filament it forms intermonomer contacts with subunits along and across the filament [38]. Due to destabilization of the monomer–monomer interface, the filaments assembled from C-terminal truncated actin appear more flexible [24]. In F-actin_−3_ changes in conformation of the DNase I-binding loop in subdomain 2 and within a segment comprising residues 227–235 of subdomain 4 were observed [26]. In addition to intermonomeric contacts, the actin C-terminus is connected to subdomains 1 and 2, as well as the nucleotide-binding site of the same monomer [25,39].

We demonstrated that binding of smooth muscle and non-muscle TM isoforms to actin alone was affected by C-terminal truncation. This observation contrasts with our earlier finding of binding of striated muscle TM to F-actin_−3_, which has been shown to be indistinguishable from binding to native actin [15]. This suggests that the contact sites on actin with the isoforms of TM used in this study differ from those of striated TM. These results are in agreement with electron microscopy studies, in which TM isoforms were shown to can assume different positions on the surface of the filament [40].

Why is skeletal TM binding neutral to the structural changes in the filament caused by C-terminal truncation? The only sequence difference between skeletal TM and non-muscle TM2 is the 27 amino acid long C-terminal segment encoded respectively by exons 9a and 9d [2]. Since the 9d-encoded C-terminus is also present in smTM and in TM5a, one might suppose that the different binding is related to this sequence. However, this is not the case, as we observed that TM1b9a binding was also sensitive to C-terminal truncation. In this chimeric protein striated muscle-specific C-terminus and N-terminus present in short non-muscle isoforms are combined. The overlap between 1a and 9a-encoded sequences differentiates skeletal TM from other TMs studied in this work. In the end-to-end overlap eleven residues from both ends of neighboring TM molecules form a complex, which is thought to correctly align and orient TM on actin [41]. Therefore, it appears that the nature of the overlap complex is important for the location of TM on actin.

Analysis of the binding data according to the linear lattice model [33] revealed additional information regarding the association of TM isoforms with actin. For each isoform of TM that was analyzed, actin truncation changed *ω*, the parameter which is linked to cooperativity between adjacent TM molecules. This supports the idea that distant conformational changes transmitted along actin participate in the cooperativity of TM binding [14,42]. The way structural alterations within the C-terminal region of actin affect TM binding depends on the isoform of TM. The decrease of the actin affinity is due to either reduced binding to an isolated site, with no adjacent binding of TM molecules, or it is due to reduced binding cooperativity. This may indicate that the specific localization of TM on the filament determines not only direct TM–actin interactions but also more distant conformational changes within the filament.

When myosin heads are bound strongly to actin, the strands of TM are shifted towards the inner domain of the filament [18,40]. Thus, an allosteric change in subdomain 4 (e.g. the segment comprising residues 227–235) associated with C-terminal truncation can contribute to the binding of TM. Differential interactions of skeletal and cardiac isoforms of TM with actin fluorescently labeled in subdomain 4 were observed by Rubenstein and collaborators [6]. Here we have shown that the cooperativity of S1-induced TM binding to truncated actin depends on the isoform of TM. The isoforms that are neutral to actin truncation in the S1-induced binding assay (smTM and TM2), share ends with the same sequences encoded by exons 1a and 9d. This suggests that the 1a9d complex present in long TMs drives a specific location on the filament, such that the distortion of actin structure does not affect myosin S1-induced binding of these isoforms. All other combinations of the ends result in TMs that are responsive to the conformational changes associated with C-terminal modification.

If the high affinity of TM to the active state were the major determinant of the cross-bridge activity, then the inhibition of the ATPase should be much reduced in the presence of the TM isoforms, which bind more easily to S1-activated F-actin_−3_, than with the isoforms, which show no sensitivity to actin truncation in S1-induced binding. The comparison of the isoforms (Fig. 6) shows no such correlation. Recently published data support our results by showing that the regulation of actomyosin activity is influenced by the sequence and flexibility of certain TM regions rather than by affinity of TM to actin in different states of activation [43,44].

In summary, isoforms of TM are able to detect conformational changes in actin. For this function a specific combination of the ends, which form an end-to-end overlap complex, is required. For binding to actin alone all combinations of sequences encoded by exons 1a, 1b, 9a, and 9d were sensitive to conformational changes in actin structure associated with C-terminal truncation, except for the overlap 1a9a found in skeletal TM. For the binding of TM in the myosin-induced state only non-muscle isoforms with overlap sequence 1a9d were insensitive to actin conformational changes. The native structure of the actin C-terminus is important for actin-myosin interactions as it keeps the filament in a more inhibited state. The increase in TM affinity to actin associated with the transition into a myosin-induced state does not seem to play a major role in this process. The data shown here support the idea that actin conformational changes can differentially drive TM isoforms to specific cellular structures.

## Figures and Tables

**Table 1:** Parameters of TM isoforms binding to native F-actin and truncated F-actin_−3_

TM	*K*_app_ (μM^− 1^)⁎		*α*^*H*^⁎		*K*_o_ (μM^− 1^)^†^		*ω*^†^	
isoform:	F-actin	F-actin_−3_	F-actin	F-actin_−3_	F-actin	F-actin_−3_	F-actin	F-actin_−3_
smTM	1.22 ± 0.04	1.01 ± 0.02	2,08 ± 0,19	2.45 ± 0.19	0.041	0.023	25.3	40.9
TM2	1.89 ± 0.07	1.36 ± 0.05	2.73 ± 0.32	2.33 ± 0.24	0.025	0.023	64.5	51.1
TM1b9a	3.80 ± 0.26	3.20 ± 0.16	1.70 ± 0.25	2.30 ± 0.26	0.108	0.082	24.1	33.2
TM5a	3.68 ± 0.69	2.46 ± 0.37	1.60 ± 0.28	2.00 ± 0.35	0.21	0.089	16.1	25.6

In this and the following tables the data obtained were averaged from 3 to 5 independent experiments done for each TM isoform; ± SE. The data in the binding isotherms in Fig. 3 were analyzed using two methods to obtain values for the affinity and cooperativity. Data obtained by fitting the experimental points to Hill equation (⁎) (Eq. 1) or to McGhee and von Hippel equation (^†^) (Eq. 2). The equations are given in Materials and methods section. Conditions: 5 mM imidazole, pH 7.0, 1 mM DTT, 150 mM NaCl, 2 mM MgCl_2_ (or 30 mM NaCl, 0.5 mM MgCl_2_ for TM5a).

**Table 2:** Parameters of myosin S1-induced binding of TM isoforms to native and truncated actin

TM isoform:	Half maximal saturation^a^	
	Native F-actin	F-actin_−3_
smTM	0.20 ± 0.02 (1.4)	0.19 ± 0.01 (1.3)
TM2	0.22 ± 0.02 (1.5)	0.22 ± 0.04 (1.5)
TM1b9a	0.15 ± 0.02 (0.9)	0.07 ± 0.01 (0.4)
TM5a	0.19 ± 0.01 (1.1)	0.08 ± 0.02 (0.5)

a Half maximal saturation represents S1/actin molar ratio required for 50% saturation of actin filaments with TM under the conditions of each experiment. The numbers were obtained by fitting experimental points to Eq. 3, as described in Materials and methods. In parenthesis-S1/TM molar ratio obtained by multiplying S1/actin by the number of actin monomers binding to 1 TM molecule (i.e. 7 for long TMs and 6 for short TMs). Conditions: 5 mM imidazole, pH 7.0, 30 mM (or 12 mM for TM5a) NaCl, 0.5 mM MgCl_2_, 1 mM DTT.

**Table 3:** Summary of the effect of actin truncation on the regulation of myosin S1 ATPase by different TM isoforms

TM isoform:	Native F-actin		F-actin_−3_	

*K*_M_^b^	*V*_max_^a^	*K*_M_^b^	*V*_max_^a^
Unregulated (34)	45.5 ± 10.7	1.01 ± 0.32	44.1 ± 9.5	0.77 ± 0.33
smTM (10)	40.2 ± 10.9	0.54 ± 0.24	41.2 ± 13.2	0.54 ± 0.27
TM2 (4)	46.0 ± 10.9	0.72 ± 0.12	47.0 ± 12.1	0.59 ± 0.33
TM1b9a (5)	49.8 ± 8.5	0.50 ± 0.12	48.2 ± 12.1	0.46 ± 0.15
TM5a (4)	47.4 ± 12.9	1.38 ± 0.49	45.5 ± 9.2	1.18 ± 0.43

The data are averages from the number of measurements given in parenthesis ± SE.

a μmol P_i_/mg S1/min.

b μM.

## Footnotes

1 In the original paper this formula has been printed with a typographical error.
